# RETRACTION: CGVD: a genomic variation database for Chinese populations

**DOI:** 10.1093/nar/gkz1105

**Published:** 2019-11-16

**Authors:** Jingyao Zeng, Na Yuan, Junwei Zhu, Mengyu Pan, Hao Zhang, Qi Wang, Shuo Shi, Zhenglin Du, Jingfa Xiao

**Affiliations:** 1 National Genomics Data Center, Beijing 100101, China; 2 BIG Data Center, Beijing Institute of Genomics, Chinese Academy of Sciences, Beijing 100101, China; 3 CAS Key Laboratory of Genome Sciences and Information, Beijing Institute of Genomics, Chinese Academy of Sciences, Beijing 100101, China; 4 University of Chinese Academy of Sciences, Beijing 100101, China

*Nucleic Acids Research*, gkz952, doi:10.1093/nar/gkz952

The authors of the above-cited article request its retraction.

The authors have not received approval from the relevant authorities to use Chinese genomic data and therefore have to suspend the CGVD database.

